# Engineered Plant-Derived Extracellular Vesicles: A Novel Strategy for Tumor-Targeted Therapy

**DOI:** 10.3390/pharmaceutics18050577

**Published:** 2026-05-07

**Authors:** Yuan Zuo, Jinying Zhang, Xinxin Wang, Bo Sun, Shuo Tian, Mingsan Miao

**Affiliations:** Academy of Traditional Chinese Medicine, Henan University of Chinese Medicine, Zhengzhou 450046, China; zzuoyuan@163.com (Y.Z.); zhang149162@163.com (J.Z.); 18836945424@163.com (X.W.); 13466144534@163.com (B.S.); tianshuo0416@163.com (S.T.)

**Keywords:** engineered plant-derived exosome-like nanovesicles, cancer, nanocarriers, drug delivery, surface modification, covalent and noncovalent modifications, artificial bionic

## Abstract

Cancer remains a leading cause of premature death worldwide, posing a significant burden due to its high incidence and mortality. Radiotherapy and chemotherapy remain the most well-established and effective modalities in the current oncological therapeutic arsenal. However, their efficacy is often limited by toxicities owing to their non-selective targeting of rapidly dividing cells and consequent damage to healthy tissues. In recent years, advances in nanomedicine and biotechnology have drawn increasing attention to plant-derived extracellular vesicles (PDEVs) as an emerging, promising strategy for cancer therapy. As novel therapeutic vehicles, PDEVs offer key advantages, including high biocompatibility and low immunogenicity. However, their clinical translation has been significantly hampered by inherent limitations, including insufficient targeting specificity, low and uncontrollable drug-loading efficiency, and challenges in large-scale production and standardization. Current research is actively focused on overcoming these drawbacks through engineering strategies, for instance, surface modification with targeting peptides or antibodies to enhance targeting, alongside optimization of production and drug-loading processes. These developments underscore the potential of PDEVs as a promising platform for next-generation targeted cancer therapeutics. This review provides a comprehensive overview of PDEVs, covering their isolation, biogenesis, physicochemical properties, and anticancer applications. While summarizing these fundamental aspects, this review focuses on engineering strategies to enhance their active targeting capacity, offering theoretical insights to support their future role in cancer treatment.

## 1. Introduction

Cancer remains a prominent and urgent challenge in global health, representing one of the leading causes of morbidity and mortality worldwide [1,2]. Current therapeutic modalities for cancer include chemotherapy, surgical intervention, immunotherapy, radiotherapy, or a combination thereof [3]. Although these approaches can effectively target and eliminate malignant cells, they are often associated with severe adverse effects, such as cardiotoxicity, neurotoxicity, and gastrointestinal and renal damage, as well as bone marrow suppression, mucositis, and alopecia, which significantly compromise patients’ quality of life [4,5,6]. To this end, there is an urgent need to develop novel strategies that can selectively target cancer cells while sparing healthy tissues. This approach is crucial for minimizing treatment-related toxicity and improving patient outcomes. Nanomedicines possess unique biological properties stemming from their nanostructures. They enhance drug solubility, stability, and targeting capability [7] while also significantly reducing side effects associated with anticancer drugs. Among various nanoplatforms applicable for clinical translation, vesicle-based nanomedicine has become one of the most widely utilized strategies. However, delivery systems based on synthetic nanovesicles still face multiple challenges in cancer treatment. Their key limitations encompass inadequate physical stability and a poor capacity to penetrate biological barriers, such as tissue and cellular membranes. Plant-derived extracellular vesicles (PDEVs) exhibit a structure analogous to synthetic nanovesicles (e.g., liposomes), comprising proteins, lipids, nucleic acids, and other bioactive components derived from donor cells. They play a crucial role in intercellular communication [8] and can be secreted by nearly all living cells. Over the past decade, PDEVs have emerged as a novel therapeutic option for a variety of diseases. Owing to their endogenous origin and capacity for intercellular transfer, PDEVs are also being explored as drug delivery vehicles for targeted therapy. Compared with conventional synthetic nanoparticles, such as liposomes, these natural carriers exhibit superior biocompatibility and minimal cytotoxicity [9]. However, issues such as immunogenicity due to interspecies variability and insufficient targeting specificity render PDEVs susceptible to short half-life and limited therapeutic efficacy, thereby constraining their utility in nanomedicine [10]. Consequently, engineering native PDEVs to overcome these limitations has garnered significant interest. Strategies such as membrane hybridization, membrane camouflage, and physical or chemical modifications can enhance targeted delivery and improve tissue uptake, thereby boosting therapeutic outcomes. Furthermore, loading PDEVs with bioactive molecules, including proteins, expression vectors, siRNAs, and DNA, can potentiate their therapeutic effects. This article systematically summarizes the application characteristics of PDEVs as novel anticancer agents. It further focuses on analyzing the engineering strategies designed to enhance their active targeting ability. The overarching aim is to provide theoretical support for expanding the therapeutic potential of engineered PDEVs.

## 2. Overview of PDEVs

### 2.1. Isolation and Purification of PDEVs

PDEVs can be isolated from diverse matrices, including tender tissues (e.g., fruits, leaves), hard tissues (e.g., rhizomes, tuberous roots), suspension cell cultures, and secretory fluids/exudates. These matrices differ substantially in cell wall architecture, water content, and polysaccharide and fiber contents, which directly influence the choice of pretreatment strategies and isolation methods. For tender tissues, cell disruption is typically achieved via low-temperature grinding or high-speed homogenization. Hard tissues, characterized by dense cell walls and high polysaccharide content, require enzymatic pretreatment using cellulase and pectinase to enhance PDEV release efficiency. In the case of suspension cell cultures, the culture supernatant can be directly collected, and cell debris is removed by low-speed centrifugation before subsequent isolation steps [11].

The isolation and purification of PDEVs represent the initial and critical step for related research and applications. Primary isolation methods include ultracentrifugation, sucrose density gradient centrifugation, tangential flow filtration, ultrafiltration, polymer-based precipitation, size-exclusion chromatography, immunoaffinity capture, and microfluidic separation techniques. Although standardized protocols for PDEV isolation are still lacking, numerous studies have comprehensively evaluated the advantages and limitations of various approaches.

For instance, ultracentrifugation is straightforward and allows high throughput, yet it is costly and time-consuming and may compromise the structural integrity of PDEVs. Polymer-based precipitation, while simple and economical, often falls short in effectively removing contaminants such as proteins and plant debris. Size-exclusion chromatography excels at preserving the integrity and homogeneity of PDEVs but requires specialized instrumentation and may result in the co-isolation of polysaccharides. Density gradient centrifugation offers high purity and is relatively low in cost; however, its lengthy processing time and limited yield hinder its potential for large-scale applications. Tangential flow filtration maintains high purity, high throughput, and structural integrity even during scale-up, though the equipment involved is expensive. Immunoaffinity capture enables highly specific and pure isolation, performing effectively even with large-volume dilute samples, yet it is both time-intensive and costly [12,13].

To address these challenges, integrated strategies that combine multiple separation techniques have shown considerable promise. For example, a recently developed approach that hybridizes gradient filtration with high-speed centrifugation has been applied to the isolation and purification of PDEVs [14]. Such engineered integrated workflows not only broaden the technical avenues and source diversity for large-scale PDEV production but also effectively preserve their therapeutic activity. This dual benefit significantly enhances the potential to advance future PDEV applications.

### 2.2. Biogenesis Pathways of PDEVs

The release of PDEVs constitutes a fundamental and indispensable process in intercellular communication. To date, at least three distinct pathways for PDEV secretion have been identified: (1) fusion of multivesicular bodies (MVBs) with the plasma membrane; (2) the exocyst-positive organelle (EXPO) pathway; (3) the vacuolar pathway [15,16,17,18]. Additionally, their release may also be associated with autophagosomes [14]. Current research primarily focuses on nanovesicles smaller than 200 nm, encompassing exosomes and some microvesicles. Microvesicles are mainly formed through outward budding of the plasma membrane [19]. Biogenesis typically initiates with the invagination of the plasma membrane, which encloses proteins, nucleic acids, and secondary metabolites into early endosomes. These early endosomes then mature into late endosomes, also referred to as MVBs, which contain numerous intraluminal vesicles. Finally, these intraluminal vesicles are released into the extracellular environment via exocytosis, at which point they are designated as exosomes [20].

Beyond the classical MVB pathway, PDEVs can also be generated through alternative pathways, including the EXPO pathway and lysosome-mediated secretion. Furthermore, their formation has been linked to vacuolar involvement. Based on their biological origin, particle size, and physical characteristics, PDEVs can be classified into three main subtypes: exosomes (diameter 30–150 nm), microvesicles (diameter 100–1000 nm), and apoptotic bodies (diameter >1000 nm). With advancing research, additional PDEV subpopulations, such as microparticles, ectosomes, and oncosomes, have been identified and categorized according to their unique biogenesis pathways and cellular origins [21].

### 2.3. Characterization of PDEVs

#### 2.3.1. Physical Characterization

According to the Minimal Information for Studies of Extracellular Vesicles (MISEV) guidelines established by the International Society for Extracellular Vesicles (ISEV), comprehensive characterization of PDEVs should include: morphological analysis, quantitative assessment of particle number and concentration, analysis of particle size distribution, quantification of total protein, lipid, and RNA content, profiling of protein composition, identification of non-protein markers, and evaluation of other functionally relevant components [22]. The morphological characterization of PDEVs is typically conducted using electron microscopy techniques, including scanning electron microscopy (SEM), transmission electron microscopy (TEM), cryo-electron microscopy (cryo-EM), and atomic force microscopy (AFM). Among these, TEM offers superior resolution compared to SEM, enabling clearer visualization of finer structural details. However, both methods require sample dehydration and fixation, which may cause deformation of PDEVs, resulting in cup- or bowl-shaped appearances under microscopy [23]. In contrast, cryo-EM does not require fixation or staining and can provide more authentic images of PDEVs, revealing a near-spherical morphology [24]. Common techniques for determining the size and surface charge of PDEVs include dynamic light scattering (DLS) and nanoparticle tracking analysis (NTA). Due to its lower resolution and inability to measure particle concentration, DLS has been progressively replaced by NTA. Characterization of various PDEVs has consistently shown negative zeta potentials, with reported values ranging from 1.5 to 49.2 mV across different samples.

#### 2.3.2. Chemical Characterization

**Figure 1:**
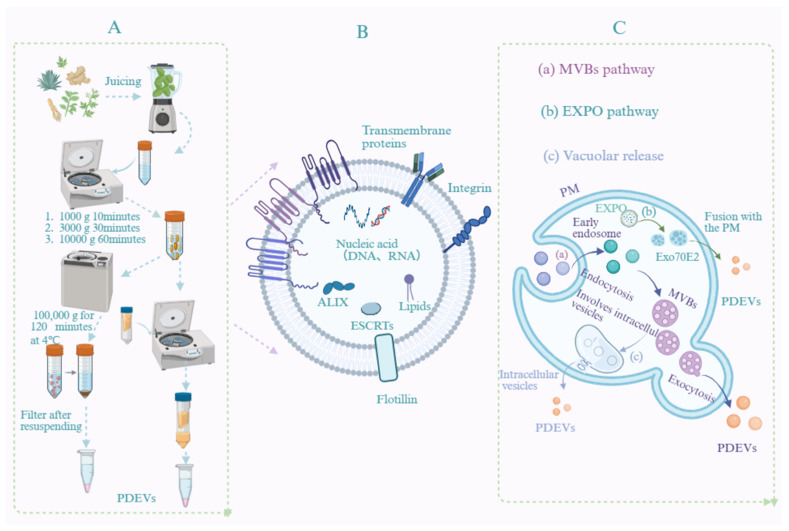
Schematic overview of PDEVs. (**A**) Isolation and extraction of PDEVs. (**B**) Characterization of PDEVs. (**C**) Biogenesis pathway of PDEVs.

PDEVs are nanoscale vesicles enclosed by a lipid bilayer, which encapsulates lipids, proteins, nucleic acids, metabolites, and other molecules, as shown in Figure 1. Lipids constitute the primary structural components of the PDEV bilayer. Lipidomic analysis reveals the lipid composition of cell membranes, including phosphatidylcholine, sphingomyelin, cholesterol, and other lipid species. This composition not only determines the structural integrity and physical properties (e.g., stiffness, fluidity) of the membrane but also participates in cell signaling and vesicle formation processes. A thorough understanding of the lipid composition and physical characteristics of natural cell membranes and extracellular vesicles is essential for the rational design of biomimetic drug delivery systems. Studies have demonstrated that the asymmetric distribution of phospholipids in native membranes, the regulatory effect of cholesterol content on membrane stiffness, and lipid–protein interactions provide important theoretical foundations for the selection of lipid components in biomimetic vesicles. Therefore, for the characterization of PDEVs, systematic identification of their lipid profiles, including the relative abundance and acyl chain composition of various phospholipids, sphingolipids, and phytosterols, should be performed using techniques such as high-resolution mass spectrometry (LC-MS/MS). Furthermore, the correlations between these lipid features and vesicle stability, as well as cellular uptake efficiency, should be evaluated [25].

PDEVs predominantly contain functional proteins associated with cytoskeletal organization, metabolic signaling, intracellular trafficking, and secretory pathways. Both membrane proteins and luminal proteins of PDEVs serve as major carriers of their biological functions. Proteomic characterization of PDEVs can reveal their biogenesis mechanisms, cell-targeting capabilities, and immunomodulatory potential. For instance, in the preparation of hybrid PDEVs, proteomic analysis can confirm that the hybrid vesicles retain the functional protein characteristics of the parent cells, which is critical for achieving homotypic targeting. Consequently, in the characterization of PDEVs, techniques such as gas chromatography–mass spectrometry, liquid chromatography–mass spectrometry, Western blotting, Coomassie Brilliant Blue assay, bicinchoninic acid assay, and label-free or labeled quantitative proteomic methods should be employed to identify the protein profiles of PDEVs, including transmembrane proteins, membrane-anchored proteins, heat shock proteins, and metabolism-related enzymes. Comparative analysis with the original plant tissues should be performed to evaluate the impact of isolation and purification processes on protein composition [26]. The integrated application of lipidomic and proteomic strategies not only facilitates the establishment of structure–activity relationships between the quality attributes and biological functions of PDEVs, but also provides a molecular-level basis for subsequent engineering modifications and the rational design of biomimetic delivery systems.

## 3. Advantages of PDEVs and Engineered PDEVs

Compared to synthetic nanoparticles, PDEVs demonstrate multiple clinically relevant characteristics and superior therapeutic advantages, including wide availability, high biocompatibility, low immunogenicity, minimal toxicity, notable stability, and targeting capability. Firstly, PDEVs can be derived from a wide range of plant sources, enabling large-scale production through simple, cost-effective preparation processes. This facilitates the generation of substantial quantities of PDEVs for therapeutic applications. Secondly, PDEVs contain natural bioactive components and exhibit high biocompatibility and low toxicity. Composed of phospholipid bilayers and endogenous biomolecules, they can be metabolized through natural physiological pathways and are generally regarded as safe and non-toxic. Moreover, they present minimal risk of carrying infectious pathogens or triggering adverse immune responses. In terms of stability, PDEVs have shown remarkable robustness. For instance, Zhang et al. [27] reported that ginger-derived exosome-like nanovesicles (GDELNs) remained highly stable in simulated gastric and intestinal fluids and demonstrated resistance to freeze–thaw cycles. These findings not only confirm their structural integrity but also support the feasibility of oral administration. Furthermore, PDEVs exhibit intrinsic targeting ability and efficient drug delivery properties, which are likely mediated by surface proteins or lipid components that promote selective uptake by specific cell types. For example, PDEVs isolated from ginger and loaded with siRNA and chemotherapeutic agents have been shown to successfully deliver their cargo to target cancer cells without causing significant damage to major organs. Notably, they also appear to mitigate side effects commonly associated with conventional chemotherapy [28].

Engineered PDEVs offer numerous significant advantages through precise modifications and functionalization, making them an ideal platform for advanced drug delivery systems. Firstly, surface-displayed targeting ligands (e.g., RGD peptide, folic acid) enable engineered PDEVs to specifically recognize diseased cells. This active targeting markedly enhances drug delivery efficiency and improves penetration across physiological barriers, such as the blood–brain barrier. Secondly, innovative drug-loading strategies enable the safe co-encapsulation of diverse therapeutics, including chemotherapeutic agents, nucleic acids, and proteins. This capability facilitates multi-mechanism combination therapies. Furthermore, surface engineering, including PEGylation, effectively prolongs systemic circulation time and reduces off-target distribution in healthy tissues. These properties, when combined, minimize toxic side effects while preserving favorable biocompatibility and low immunogenicity. Ultimately, this modular and customizable design paradigm allows engineered PDEVs to be flexibly adapted to diverse therapeutic requirements, demonstrating considerable translational potential for achieving precise drug delivery, enhanced therapeutic efficacy, and improved safety profiles.

## 4. The Role of Natural PDEVs in Cancer Suppression

**Figure 2:**
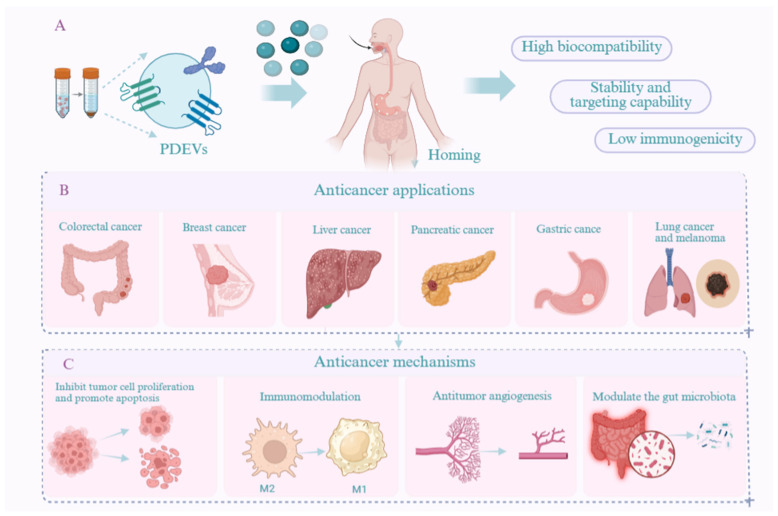
(**A**) Advantages, (**B**) applications in cancer therapy, and (**C**) therapeutic mechanisms of PDEVs.

PDEVs are nanoscale vesicles with a lipid bilayer structure secreted by plant cells. They encapsulate various bioactive molecules, including lipids, proteins, and nucleic acids. These components serve as information carriers that mediate intercellular and interspecies communication through material transfer and molecular signaling [29]. PDEVs can not only transport their own bioactive components but also serve as delivery vehicles for other active substances or therapeutic drugs. Due to their high biocompatibility, low toxicity, targeting capacity, and remarkable bioactivity, PDEVs have attracted widespread research interest. Numerous studies have demonstrated that PDEVs exhibit significant anti-tumor activity through multiple mechanisms. For instance, Huang et al. [30] reported that exosome-like nanoparticles derived from Centella asiatica were effectively internalized by HepG2 cells. These internalized vesicles induced increased ROS production, mitochondrial damage, cell cycle arrest, elevated CASP3 activity, and dysregulation of Bax and Bcl-2 protein expression, ultimately leading to tumor cell apoptosis. Similarly, Morus nigra L-derived exosome-like nanoparticles were found to induce G0/G1-phase cell cycle arrest and promote apoptosis in Hepa1-6 cells. They also triggered a surge in intracellular ROS levels and significantly suppressed the proliferation and migration of liver cancer cells [31]. These findings illustrate that PDEVs can directly inhibit tumor proliferation and induce apoptosis by regulating the cell cycle (e.g., G2/M-phase arrest) or activating apoptotic pathways (e.g., caspase-3/9, Bax upregulation, Bcl-2 downregulation). PDEVs also exert anti-tumor effects by inhibiting cancer cell metastasis and invasion. In a study by Yang et al. [32], exosome-like nanoparticles derived from Brucea javanica were shown to deliver functional miRNAs to 4T1 cells, significantly impeding tumor growth and metastasis by modulating the PI3K/Akt/mTOR signaling pathway and promoting ROS- and caspase-mediated apoptosis. Furthermore, these nanoparticles inhibited the secretion of vascular endothelial growth factor, contributing to anti-angiogenesis within the tumor microenvironment. In vivo experiments confirmed that Brucea javanica-derived PDEVs suppressed tumor growth, metastasis, and angiogenesis in a mouse model of breast cancer while maintaining a high safety profile. Additionally, PDEVs have been demonstrated to modulate the tumor immune microenvironment. For example, exosome-like nanoparticles isolated from Artemisia annua inhibited tumor growth and enhanced anti-tumor immunity in a mouse model of lung cancer, primarily by remodeling the tumor microenvironment and reprogramming tumor-associated macrophages (TAMs). The researchers identified plant-derived mitochondrial DNA (mtDNA) encapsulated in PDEVs as the key effector molecule. Upon internalization by TAMs, this mtDNA activates the cGAS-STING pathway. Consequently, this activation drives the repolarization of pro-tumor macrophages toward an anti-tumor phenotype [33]. This study highlights the ability of PDEVs to achieve anti-tumor effects by modulating the tumor microenvironment. PDEVs can also induce polarization of pro-inflammatory M1 macrophages while suppressing immunosuppressive M2 macrophages by inhibiting the TLR/NF-κB pathway. Moreover, certain PDEVs contain antioxidants such as superoxide dismutase and glutathione, which scavenge reactive oxygen species and alleviate oxidative stress in tumors, as summarized in Table 1 and Figure 2.

## 5. The Applications of Engineered PDEVs in Cancer Treatment

Similar to exosomes from other sources, PDEVs can deliver bioactive substances to target cells, thereby facilitating intercellular communication. Owing to this key function, they are emerging as a promising approach for cancer therapy. However, fully exploiting their therapeutic benefits remains challenging due to their low bioactive molecule content and lack of precise targeting to cancer sites. To address these limitations, engineered PDEVs have been developed to enhance tumor-targeted delivery. Active targeting strategies are essential to achieve such specificity, and several engineering approaches have been explored, including drug loading, membrane hybridization, membrane camouflage, surface modification, and artificial biomimicry [49]. These strategies are designed to significantly improve the therapeutic potential of PDEVs. Among them, surface modifications and membrane hybridization primarily aim to enhance stability and targeting efficiency, while drug loading is generally employed to improve protective efficacy and delivery. The engineering of PDEVs remains in its early stages. Nevertheless, synergistically applying multiple engineering techniques, a strategy inspired by artificial nanoparticle research, paves a promising path for future development.

### 5.1. Drug Delivery Vehicle

PDEVs not only serve as messengers for intercellular and interspecies material exchange and information communication but also function as natural active nanocarriers. They possess the capacity to encapsulate and efficiently deliver functional therapeutic molecules, such as small-molecule drugs, nucleic acids, and proteins, to tumor cells. Compared to synthetic nanocarriers, PDEVs exhibit superior biocompatibility, oral safety, and an exceptional ability to cross biological barriers, making them particularly promising for drug delivery. For instance, Wang et al. [50] demonstrated that grapefruit-derived nanovesicles (GDNs) act as highly efficient nanocarriers for therapeutic agents. Moreover, PDEVs show remarkable resilience in highly acidic gastric environments and remain stable against intestinal proteolytic enzymes [51], further supporting their potential as robust oral delivery systems. They have significant potential in delivering poorly soluble drugs and natural biomolecules. While the functionalization of artificial nanovesicle structures has been extensively studied, the engineering of PDEVs may pose both a challenge and an intriguing opportunity for the development of personalized therapeutic vectors.

#### 5.1.1. Small-Molecule Drugs

Traditional anticancer drugs are often limited by drug resistance, insufficient targeting, and a lack of specificity. In this context, PDEVs have emerged as a novel and promising drug delivery platform for cancer therapy. These naturally derived nanovesicles exhibit outstanding biocompatibility, targeted delivery capacity, and the ability to encapsulate therapeutic agents, positioning them at the forefront of innovative cancer treatment strategies. To achieve optimal delivery, several factors need to be addressed: enhancing encapsulation efficiency, ensuring nanoparticle structural integrity, and maintaining the drug molecule’s bioactivity. Multiple techniques can be employed to encapsulate drugs within PDEVs, encompassing co-incubation, electroporation, sonication, click chemistry, freeze–thaw, osmotic shock, etc. [52]. Zhang et al. [53] demonstrated that doxorubicin (Dox) could be efficiently loaded into GDELNs via ultrasonication. Compared with commercial liposomal Dox, the GDELN-Dox system exhibited superior pH-dependent drug release behavior. Furthermore, GDELNs-Dox specifically delivered Dox to Colon-26 tumor cells, leading to significantly enhanced chemotherapeutic efficacy in suppressing tumor growth in vivo. Beyond Dox, GDELNs also serve as an effective delivery vehicle for curcumin. The resulting complex exerted a notable protective effect against ulcerative colitis by modulating host serum metabolite levels and the gut microbiota [54]. Cisplatin-loaded ginseng-derived exosome-like nanovesicles (GELNs) effectively targeted tumor sites, inhibited the proliferation and migration of U-87MG cells, and promoted their apoptosis. Notably, this system required 12.66-fold less cisplatin than free cisplatin to achieve equivalent cytotoxic effects. In U-87MG tumor-bearing mouse models, the system efficiently targeted tumor tissue and demonstrated significant therapeutic efficacy [55]. These findings indicate that this strategy maintains potent anti-tumor activity while reducing cisplatin dosage, suggesting that it is an effective alternative to conventional chemotherapy in clinical practice. Furthermore, loading 5-fluorouracil into bitter melon-derived exosome-like nanovesicles produced a dual enhancement: it not only potentiated the anticancer efficacy of 5-FU but also counteracted chemoresistance in oral squamous cell carcinoma. The synergistic mechanism appears to involve downregulation of the key inflammatory protein NLRP3 during drug delivery, which likely contributes to reversing drug resistance [46]. In addition to medicinal plants, edible plants can serve as sources of exosome-like nanovesicles for drug loading. For instance, GDNs can encapsulate various anticancer agents such as methotrexate, paclitaxel, and sodium thiosulfate, demonstrating promising therapeutic outcomes across multiple disease models. In a DSS-induced murine colitis model, methotrexate-loaded GDNs reduced drug toxicity and enhanced therapeutic efficacy compared to free methotrexate. This dual effect suggests that GDNs have potential both as immunomodulators to maintain intestinal macrophage homeostasis and as an oral delivery platform for small-molecule drugs against human inflammatory diseases [50,56,57]. Furthermore, sodium thiosulfate-loaded GDNs were shown to alleviate vascular calcification and improve biocompatibility by promoting M2 macrophage polarization, suppressing inflammation, and inhibiting the bone–vascular axis. These findings collectively highlight the versatile potential of GDNs as multifunctional drug carriers. Moreover, indocyanine green loaded into aloe-derived exosome-like nanovesicles exhibited high stability under heating and in serum. This drug delivery system effectively killed melanoma cells and suppressed tumor growth even after storage, outperforming both free indocyanine green and its liposomal formulation. It also demonstrated significant skin penetration ability in mice, indicating promise for non-invasive transdermal drug delivery [58].

#### 5.1.2. Nucleic Acid and Protein Drugs

Developing safe and efficient nanocarriers for gene delivery remains a critical challenge for advancing gene therapy. Currently available viral and non-viral vectors carry potential risks, such as immunogenicity and toxicity [59]. In recent years, PDEV-based gene nanocarriers have emerged as a promising strategy for targeted delivery and enhanced therapeutic efficacy, owing to their remarkable stability under various environmental conditions [60]. Among these, PDEV-based carriers have been extensively studied for the delivery of therapeutic RNAs to treat a range of diseases. For example, GDELNs have shown excellent targeting capability as a novel drug delivery system. These nanovesicles can be efficiently loaded with small interfering RNA targeting the CD98 gene (siRNA-CD98). Leveraging their natural homing ability, they specifically accumulate in colon tissues following oral administration. Upon reaching the target site, the vesicles are effectively internalized by intestinal cells, releasing siRNA-CD98 and mediating highly efficient silencing of the CD98 gene, offering a novel therapeutic strategy for inflammatory bowel disease and related conditions [61]. GELNs loaded with miR-182-5p demonstrate significant therapeutic efficacy in an acute lung injury model. These nanovesicles precisely deliver miR-182-5p to pulmonary cells, where they suppress NOX4 expression and downregulate its downstream protein Drp-1, ultimately inhibiting NLRP3 inflammasome activation. This cascade effectively blocks key signaling pathways of oxidative stress and pyroptosis, markedly alleviating pulmonary inflammation and tissue damage at both cellular and organismal levels [62]. Broccoli-derived exosome-like nanovesicles exhibit unique bioprotective properties. When loaded with plant-derived small RNAs (e.g., miR159a, miR159b-3p, miR166b-3p, miR403-3p), their lipid bilayers provide robust shielding from the external environment, significantly enhancing resistance to degradation by ribonucleases (RNases) in blood and tissue fluids [63]. This protective capacity ensures the structural integrity and bioactivity of functional small RNAs during delivery, establishing a foundation for their regulatory functions.

**Figure 3:**
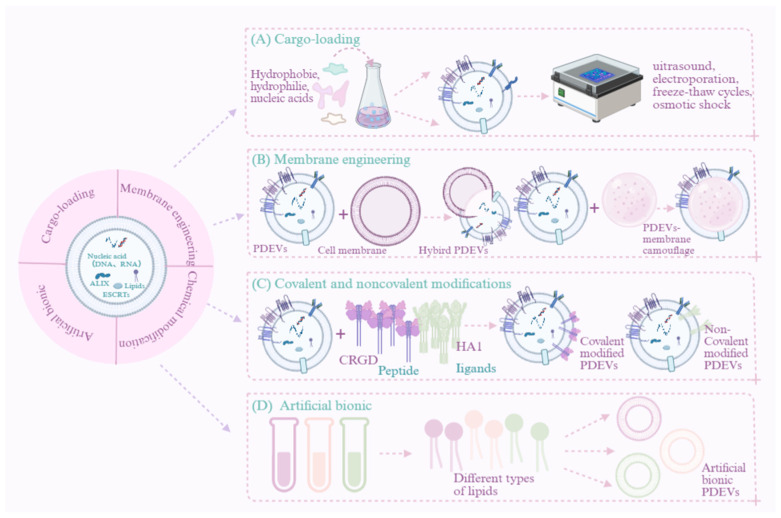
The efficacy of engineered PDEVs in cancer therapy. (**A**) Drug carriers. (**B**) Membrane hybridization and membrane modification. (**C**) Covalent and noncovalent modifications. (**D**) Artificial bionics.

PDEVs also serve as effective carriers for protein-based therapeutics. GELNs loaded with infliximab exert anti-inflammatory effects by blocking the NLRP3 inflammasome [64], while GDNs encapsulating HSP70 efficiently activate tumor-specific immune responses in the colon [65] (Table 2 and Figure 3). As an emerging platform for nucleic acid drug delivery, PDEVs combine natural origin, excellent biocompatibility, targeting capability, and unique bioprotective functions, demonstrating considerable potential. By integrating the “natural intelligence” of plants with the precision of nucleic acid therapeutics, they may not only overcome current technical barriers in drug delivery but also open a new “green” pathway for future disease treatment, nutritional intervention, and precision medicine.

### 5.2. Membrane Hybridization and Camouflage

Membrane hybridization technology is a highly efficient strategy for engineering PDEVs. This approach enables the safe, controlled use of their intrinsic biological functions without compromising structural integrity or bioactivity. By incorporating specific targeting molecules, this technology allows PDEVs to achieve high-precision recognition and detection of other lipid nanoparticles, demonstrating significant potential in drug delivery, molecular imaging, and diagnostics. For instance, GDELNs were decorated with cholesterol-anchored FA-3WJ and successfully loaded with survivin siRNA. This approach enabled efficient targeted delivery and gene knockdown with low cytotoxicity, ultimately leading to significant tumor growth suppression [28]. A hybrid complex, formed by fusing GELNs with autologous tumor membranes, enhances dendritic cell (DC) phagocytosis of tumor antigens and promotes DC maturation via the TLR4 pathway. This process robustly activates tumor-specific cytotoxic T lymphocytes, strengthening the adaptive immune response and significantly suppressing the recurrence and metastasis of both subcutaneous and orthotopic tumors. Moreover, it induces long-term immunoprotective effects and prolongs survival [76]. Folate-coated GDNs, administered intranasally, efficiently deliver miR-17 to brain tumors and enhance their cellular uptake. By downregulating MHC-I expression on GL-26 cells, they activate natural killer (NK) cells, significantly delaying glioblastoma progression in mice [77]. Furthermore, a bacterial plant hybrid vesicle constructed by fusing spinach-derived exosome-like nanovesicles with outer membrane vesicles from E. coli can target tumors following systemic administration. Through chloroplast-mediated photodynamic effects, it induces tumor destruction. This process releases tumor antigens and activates dendritic cells (DCs), thereby eliciting a potent tumor-specific CD8^+^ T-cell response. Consequently, this cascade reverses the immunosuppressive tumor microenvironment, leading to effective suppression of tumor growth and metastasis [78]. The same plant-derived nanovesicle can also be combined with NK cell membranes to form plant–chemical NK cells (PC-NKs). These PC-NKs home to tumor tissue and utilize catalase from thylakoids to deeply reprogram the tumor immune microenvironment, significantly enhancing their direct tumor-killing capacity while synergistically activating systemic anti-tumor immunity [79].

### 5.3. Covalent and Noncovalent Modifications

In the engineering of PDEVs, covalent and noncovalent modifications represent two key chemical strategies for functionalization. Covalent modification involves the formation of stable chemical bonds between active small molecules or multifunctional nanomaterials and surface functional groups on the vesicles, enabling durable regulation of their properties. For example, (1) conjugation of the aptamer HA1 to GDNs significantly enhanced their targeted distribution and cellular uptake in breast cancer tumors, leading to effective tumor growth inhibition [80,81]. (2) Similarly, conjugation with LA1 and Psi-LA1 aptamers enabled efficient tumor-targeted distribution, suppressed tumor growth, and maintained good biosafety [82]. (3) CRGD-targeted, DOX-loaded orange-derived exosome-like nanovesicles enhanced drug accumulation and penetration in ovarian cancer. This synergistically exerts anti-proliferative and anti-angiogenic effects while reducing degradation and inflammatory responses [83]. (4) Heparin-cRGD-modified lemon-derived nanovesicles successfully delivered DOX with excellent tumor targeting, long circulation, and biosafety. They exerted synergistic anti-tumor effects through pro-apoptotic, anti-proliferative, and anti-angiogenic mechanisms [84].

Noncovalent modification, in contrast, relies on weak interactions such as hydrophobic effects or receptor–ligand binding to achieve temporary assembly of functional molecules or membrane integration, offering reversibility and favorable biocompatibility. For instance, modification with DSPE-PEG-RVG ligands on aloe, Asparagus falcatus, and kudzu-derived exosome-like nanovesicles improved drug-loading efficiency, stability, and leakage prevention. This system facilitated targeted drug accumulation in tumors, effectively inhibiting cancer cell growth and migration [85,86]. PDEVs engineered through covalent and noncovalent modifications exhibit significantly enhanced targeting and delivery capabilities, drug-loading stability, and biocompatibility. This engineering strategy successfully integrates the innate low immunogenicity of natural vesicles with the programmable functionality of artificial modifications, providing a highly efficient and synergistic platform for precision drug delivery.

### 5.4. Biomimetic and Hybrid Engineering Strategies

PDEVs are regarded as ideal drug delivery carriers in preclinical studies for various diseases due to their unique endogenous biological properties. However, their clinical translation faces several challenges, including low production yield and difficulties in controlling residual impurities. More importantly, the structure and composition of PDEVs exhibit significant heterogeneity and variability, influenced by factors such as plant source, growth environment, and extraction methods, resulting in substantial batch-to-batch inconsistencies. Therefore, there is an urgent need to develop a robust, scalable biomimetic preparation strategy to construct structurally defined, functionally controllable artificial PDEV nanoparticles, thereby fully realizing their clinical potential. For instance, Qiao et al. [34] modified GDELNs with Pd–Pt nanosheets, significantly increasing intracellular ROS levels while downregulating the expression of pro-inflammatory cytokines TNF-α and IL-6. In another study, lemon-derived exosome-like nanovesicles were modified with cRGD and reconstructed via ultrasonic emulsification. This engineered system enhanced drug penetration across the blood–brain barrier, enabling targeted DOX delivery that suppressed glioblastoma growth and improved therapeutic outcomes [87] (Table 3 and Figure 3).

Owing to their excellent biocompatibility, low immunogenicity, and potential for scalable production, PDEVs have emerged as a promising nanomedicine platform, potentially surpassing animal-derived extracellular vesicles [88]. To fully exploit their therapeutic potential, engineering strategies such as “disassembly–reconstitution” have been developed to longitudinally regulate plant lipid composition and achieve efficient drug loading. This approach not only enhances the targeting specificity, bioavailability, and cellular uptake of PDEVs but also significantly improves their therapeutic efficacy. Innovative engineering methods now allow PDEVs to co-load diverse therapeutics, including chemotherapeutic agents, nucleic acids, and proteins. This capability to create modular and programmable nanocarriers positions PDEVs as a leading direction in nanomedicine, as shown in Table 4.

## 6. Summary and Outlook

Engineered PDEVs represent an emerging nanobiotechnology platform demonstrating unprecedented potential in cancer therapy. Through precision engineering strategies, including covalent conjugation, membrane fusion, and physical drug loading, targeting moieties, therapeutic drugs, and functional nucleic acids can be successfully integrated into these natural carriers. This integration confers capabilities beyond native functions: specific tumor tissue accumulation, penetration of complex physiological barriers, and stimulus-responsive controlled drug release. These modifications effectively overcome the core challenges of conventional nanomedicines, including poor targeting specificity, high systemic toxicity, and the low biocompatibility of synthetic carriers. Concurrently, they successfully transform the inherent advantages of plant exosomes, such as their biosafety, low immunogenicity, and potential for scalable production, into clinically viable features. Compared to synthetic nanoparticles, engineered PDEVs exhibit multiple structural and functional advantages. Their endogenous lipid and biomolecular composition ensures excellent biocompatibility and high bioavailability. The nanoscale size enables efficient penetration across biological barriers, particularly in tumor regions, where tissue permeability is enhanced. Additionally, naturally occurring transmembrane and membrane-anchored proteins provide specific cell-targeting capabilities. Beyond these biological advantages, PDEVs avoid ethical concerns associated with animal-derived exosomes and offer scalable manufacturing potential, presenting broad prospects for clinical translation in cancer therapeutics.

Nevertheless, the translation of PDEVs from fundamental research into widespread clinical application faces formidable challenges, primarily about scalability, standardization, and quality control. With respect to drug-loading efficiency and capacity, current physical loading methods, such as electroporation, are operationally simple but are constrained by cargo leakage and inadequate long-term stability. In the context of large-scale production and quality assurance, the intrinsic heterogeneity of PDEVs constitutes a major barrier to clinical translation, manifested as a “triple heterogeneity”: broad particle size distribution, batch-to-batch variability in bioactive components, and inconsistent biological functionality. This heterogeneity originates from multiple sources, including differences in plant species and cultivars, fluctuations in growth environments and agronomic practices, and variations inherent to extraction and preparation methods. Collectively, these factors lead to poor reproducibility of research findings, unpredictable therapeutic outcomes, and regulatory challenges. To address these issues, a full-chain standardization strategy is urgently required. At the raw material and production levels, the adoption of tissue culture technology combined with a TIBS is recommended to ensure homologous sourcing of plant materials and enable non-destructive, continuous production. At the characterization level, adherence to international guidelines such as MISEV2023 [97] is essential for systematic assessment of core quality attributes, including particle size and zeta potential. Furthermore, drawing on Chinese group standards (T/CIET 1720-2025 [98], T/SHRH 075-2025 [99]), a tiered regulatory framework may be considered, wherein simplified approval procedures apply to PDEVs intended for use as food supplements or cosmetic ingredients, whereas those intended for therapeutic applications must strictly comply with biological product standards and cGMP requirements. Regarding in vivo behavior and safety, engineering-modified PDEVs display increasingly complex pharmacokinetic profiles, including biodistribution and clearance pathways. Their long-term biosafety and potential immunogenicity risks, therefore, warrant systematic evaluation.

In light of these challenges, future research must focus on achieving breakthroughs in core areas with actionable solutions. Technologically, efforts should be directed toward developing next-generation efficient and controllable drug-loading strategies, such as microfluidics-enabled high-throughput self-assembly or genetic engineering of parental plant cells to produce “pre-loaded” functional PDEVs. Regarding scalability, the periodic immersion bioreactor system should be actively adopted and optimized. By precisely regulating culture parameters, this system has enabled standardized and continuous production of various PDEVs from medicinal and edible plants, achieving substantially improved yields while reducing energy consumption to 60% of that of conventional methods [100]. With respect to standardization and translation, a full-chain quality control system spanning from production sources to final products is urgently needed. This should encompass adherence to international guidelines such as ISO/TS 21418, as well as the active implementation of regional standards such as China’s first General Requirements for Plant-Derived Vesicle Cosmetic Ingredient Products (T/SHRH 075-2025). For the regulatory pathway, a “tiered regulation” mechanism is recommended: simplified approval procedures may apply to vesicle-based products intended as food supplements or cosmetic ingredients, whereas medicinal PDEVs intended for invasive applications such as injection must strictly comply with biological product standards and cGMP requirements [101]. Ultimately, a multidisciplinary collaborative innovation ecosystem integrating materials science, nanotechnology, bioinformatics, immunology, and clinical medicine is essential to systematically address these scientific and technological challenges. This endeavor is not merely to advance engineered PDEV development per se, but to fully unleash their substantial clinical potential, transforming this promising laboratory concept into next-generation, precise, safe, and highly effective therapeutic modalities that can truly benefit the vast population of cancer patients.

## Figures and Tables

**Table 1:** The role of PDEVs in cancer therapy.

Mechanisms	Plant	Targeted Cells	Diseases	Mechanisms	References
Direct inhibition of cancer cell proliferation	*Centella asiatica*	HepG2	Liver cancer	Induce ROS elevation, mitochondrial damage, cell cycle arrest, and apoptosis.	[30]
	*Morus nigra* L.	C57BL/6J, Hepa1-6	Liver cancer	Induce cell cycle arrest and apoptosis, and suppress cancer cell proliferation and migration.	[31]
	Asparagus	Hep G2	Liver cancer	Inhibit tumor growth without side effects.	[34]
	Ginseng	C6	Glioma	Induce apoptosis.	[35]
	Bitter melon	4T1, MCF-7, MCF-10A	Breast cancer	Elevate ROS production and disrupt mitochondrial function to inhibit tumor cell growth.	[36]
	Ginger	MDA-MB-231	Breast cancer	Induce apoptosis and cell cycle arrest, and exhibit anti-metastatic activity.	[37]
	Curcumin	SKBR3	Breast cancer	Induce apoptosis in SKBR3 cells, increase the Bax/Bcl-2 ratio, and downregulate cyclin D1 and CDK4 expression.	[38]
	*Moringa oleifera*	Hela	Cervical cancer	Downregulate Bcl-2 expression and reduce mitochondrial membrane potential.	[39]
	Tea flowers	MCF-7, 4T	Breast cancer	Inhibit tumor growth.	[40]
	Lemon	AGS, BGC-823, SGC-7901	Gastric cancer	Induce S-phase cell cycle arrest and trigger apoptosis through ROS generation in vitro.	[41]
Inhibition of cancer cell metastasis and invasion	*Brucea javanica*	4T1, HUVECs	Breast cancer	Inhibit tumor growth, metastasis, and angiogenesis.	[32]
Modulation of the tumor microenvironment	Artemisia	C57/BL6	Lung cancer	Promote the transition of tumor-associated macrophages toward an anti-tumor phenotype.	[33]
	Ginseng	C57/BL6	Melanoma	Inhibit M2-like polarization of macrophages.	[42]
	*Platycodon grandiflorum*	4T1, A549	Breast cancer	Promote M1 polarization of tumor-associated macrophages.	[43]
Anti-inflammatory and antioxidant properties	Fingerroot	HT-29, HCT116	Colorectal cancer	Disrupt redox homeostasis in cancer cells.	[44]
	Tea leaves	RAW 264.7	Colorectal cancer	Downregulate pro- and anti-inflammatory cytokine production.	[45]
	Bitter melon	Oral squamous cell carcinoma	Oral squamous cell carcinoma	Downregulate NLRP3/IL-1β expression in mouse tumors.	[46]
Modulation of the gut microbiota	Artemisiae Argyi	HT-29	Colorectal cancer	Restore gut barrier integrity while reversing microbiota dysbiosis.	[47]
	Tea leaf	A549	Lung cancer	Promote apoptosis and microbiota modulation.	[48]

**Table 2:** Applications of PDEVs as drug carriers in cancer.

Drug Type	Plants	Drugs	Method	Efficiency	Disease	Effect	Reference
Small-molecule drugs	Ginger	DOX	Sonication	95.9%	Colon cancer	Target inhibition of cancer cell growth	[53]
		Curcumin	Sonication	94.0%	Ulcerative colitis	Enhance therapeutic potential	[54]
		6-Shogaol	Sonication	89.1%	Ulcerative colitis	Modulate the gut microbiota composition	[66]
		Folic acid	Sonication	/	Ulcerative colitis	Reduce iron overload in a hereditary model	[67]
	Ginseng	Cisplatin	Sonication	/	Ovarian cancer	Target tumors to suppress proliferation and migration while inducing apoptosis	[55]
	Bitter Melon	5-Fluorouracil	Sonication	/	Tongue cancer	Reduce drug resistance in oral squamous cell carcinoma	[46]
	Grapefruit	Methotrexate	Co-incubation	/	Colon cancer	Reduce the toxicity of methotrexate	[56]
		Paclitaxel	Sonication	/	Colon cancer	Inhibit tumor growth	[50]
		Sodium thiosulfate	Co-incubation	/	Vascular calcification	Promote the polarization of M2 macrophages	[57]
	Avocado	Berberine	Freeze–thaw	/	Atherosclerosis	Rapidly internalized by macrophages	[68]
	Aloe Vera	Indocyanine green	Co-incubation	/	Melanoma	Inhibit the growth of melanoma	[58]
	Lemon	DOX	Sonication	/	Breast cancer, colorectal cancer	Enhance drug efficacy and improve targeting specificity	[69]
	Tomato	Curcumin	Sonication	/	Colorectal cancer	Inhibit the expression of IL-1β and IL-6	[70]
		Calcitriol	Sonication	47.3%	Colorectal cancer	Potentiate calcitriol’s anticancer effect	[71]
	Grape	Metformin, doxorubicin, tamoxifen	/	/	Breast cancer	Promote tumor cell apoptosis by elevating intracellular ROS levels	[72]
	Kiwi	Sorafenib	/	/	Liver cancer	Reduce dose-related toxicity while promoting liver targeting and uptake	[73]
	*Citrus reticulata* Blanco cv.	Tangeretin	/	/	Diabetes	Enhance anti-inflammatory and antioxidant effects	[74]
Nucleic acid drugs	Ginger	siRNA-CD98	Sonication	61 %	Colorectal cancer	Target the colon and mediate CD98 gene suppression	[61]
	Ginseng	miR-182-5p	Electrotransfer	/	Sepsis-associated acute lung injury	Target NOX4/Drp-1/NLRP3 signaling, ameliorating the disease	[62]
	Broccoli	miR159a, miR159b-3p, miR166b-3p, miR403-3p	Transfection	/	Colorectal cancer	Enhance resistance to RNase degradation	[63]
	Acerola	miR-340	Co-incubation	60%	Colorectal cancer	Target gene suppression in the small intestine	[75]
Protein drugs	Ginger	Infliximab	Sonication	61.3%	Colorectal cancer	Gastrointestinal stability and colon-targeted delivery	[64]
	Grapefruit	HSP70	Co-incubation	/	Colorectal cancer	Activate a colon tumor-specific response	[65]

Note: “/” indicates that no result was found.

**Table 3:** Application of engineered PDEVs in cancer therapy.

	Plant	Method	Model	Advantages	Mechanism	Reference
Membrane hybridization	Ginger	Cholesterol-anchored FA-3WJ decoration	KB tumor xenograft model	Low cytotoxicity with targeted survivin siRNA delivery and gene knockdown	Inhibit tumor growth.	[28]
	Ginseng	Membrane hybridization with resected autologous tumor-derived membranes	Breast cancer	Enhanced dendritic cell phagocytosis of autologous tumor antigens	Potentiate tumor-specific immunity, preventing recurrence and metastasis.	[76]
	Grapefruit	Folate-coated and miR-17-loaded	Brain tumor	Enhanced targeting efficiency toward brain tumor cells	Suppress MHC-I expression on GL-26 tumor cells.	[77]
	Spinach	Hybridized with outer membrane vesicles derived from E. coli MG1655	Colorectal cancer	Enhanced tumor cell targeting with an initiated immune response	Prevent tumor development and metastasis.	[78]
		NK cell membrane-hybridized	/	Activate NK cells	Tumor-targeted with enhanced NK cell-mediated cytotoxicity.	[79]
	Purple cabbage	Freeze–thaw fusion with collagen I-targeting peptide-loaded liposomes.	Heart failure	Precise delivery and efficient accumulation	Anti-inflammatory, anti-fibrotic, pro-repair via inhibition of overactivated PI3K-AKT-mTOR.	[80]
	Blex	Chemical conjugation modified the vesicle surface with cyclic RGD peptide.	Breast cancer	Enhanced cellular uptake and cytotoxicity	Active targeting.	[81]
	Watermelon	Complexed with G3 PAMAM/miR-146a mimic.	Ovarian cancer	Overcome key issues of conventional RNA delivery: low bioavailability, poor loading capacity, and scalability hurdles	MiR-146a exerts its tumor-suppressive effects primarily through anti-angiogenic activity.	[82]
Membrane camouflage	Spinach	Chondrocyte membrane-camouflaged PDEVs	Osteoarthritis	Increased intracellular ATP and NADPH levels	Elevate intracellular ATP and NADDPH levels with improved anabolism in degenerative chondrocytes.	[83]
Covalent modification	Grapefruit	Aptamer HA1-conjugated	Breast cancer	Enhanced cellular uptake and targeted tumor distribution	Inhibit tumor growth.	[84]
		Aptamer LA1- and Psi-LA1-conjugated	Colon cancer	Targeted tumor distribution with favorable biosafety	Inhibit tumor growth.	[85]
	Orange	CRGD-modified and DOX-loaded	Ovarian cancer	Enhanced tumor accumulation and penetration with reduced degradation and inflammation	Anti-proliferative and anti-angiogenic effects against tumors.	[86]
	Lemon	Heparin-cRGD-modified and DOX-loaded	Ovarian cancer	Enhanced tumor targeting, prolonged retention, and favorable biosafety	Promote apoptosis with anti-proliferative and anti-angiogenic effects.	[34]
Noncovalent modification	Aloe vera	DSPE-PEG-RVG-modified	Breast cancer	Enhanced loading efficiency, stability, and leakage prevention for competitive drugs	Suppress tumor cell growth and migration with enhanced drug accumulation at tumor sites.	[87]
	Asparagus	PEG modification	Liver cancer	Enhanced circulation longevity and tumor targeting	Inhibit tumor growth.	[87]
	Puerariae Radix	DSPE-PEG-RVG-modified	Parkinson	Enhanced blood–brain barrier penetration and dopaminergic neuron targeting	Improve autonomic behavior in mice.	[88]
Artificial bionic	Ginger	Pd–Pt nanosheet-modified	/	Increase the expression level of ROS	Reduce the expression levels of TNF-α and IL-6.	[89]
	Lemon	CRGD modification and reconstruction via ultrasonic emulsification	Glioblastoma	Promote penetration of the blood–brain barrier	Target delivery of DOX and inhibition of tumor growth.	[90]
	Grapefruit	Heparin-targeted, DOX-loaded nanoparticle modification	Neuroglial tumor	Increase the drug-loading capacity and penetrate the blood–brain barrier	Enhance the absorption of drugs in tumors and anti-angiogenesis.	[91]
	Synthetic lipids	Fusion of synthetic lipids with colorectal cancer cell membranes	Colorectal cancer	Significantly enhanced homotypic targeting efficiency and biocompatibility	Homotypic tumor targeting and efficient uptake via surface-integrated CRC membrane proteins.	[26]

**Table 4:** Comparison of key characteristics of engineering strategies for PDEVs.

	Physical Loading	Membrane Hybridization	Membrane Camouflage	Covalent Modification	Noncovalent Modification
Advantages	Mature process, easy scale-up; suitable for industrial production; TIBS system enables standardized manufacturing.	High functional integration confers novel bioactivities while preserving plant-derived miRNAs/polyphenols.	Enables immune evasion and reduces macrophage uptake; achieves cell-selective uptake via homologous targeting.	Highest conjugation stability; controllable modification density; amenable to standardized protocols; well-established regulatory experience in ADC and PEGylated liposome fields.	Mild conditions preserve vesicle integrity; simple and efficient operation; no immunogenicity risk associated with covalent bonds.
Limitations	Susceptible to cargo leakage; electroporation/sonication may induce vesicle membrane damage; poor long-term storage stability.	Batch variability of membrane sources; composition-dependent fusion efficiency; phase separation risk (long-term storage).	Complex extraction; source-specific optimization; scalability challenges.	Chemical reaction conditions may disrupt surface protein and lipid conformation; introduction of non-natural linkers may increase immunogenicity; multi-step purification adds cost.	Electrostatic adsorption and hydrophobic insertion offer lower stability than covalent bonds; avidin/streptavidin, as exogenous proteins, may pose immunogenicity risks.
Efficiency	High loading efficiency via electroporation/sonication.	High lipid fusion efficiency, but plant vesicle heterogeneity may compromise fusion consistency.	High efficiency; preserves the full functionality of membrane proteins.	Stoichiometric ratio optimization required.	High efficiency; simple operation; no complex chemical reactions; preserves native molecular conformation.
Stability	Prone to cargo leakage; physical adsorption is environmentally sensitive.	Relatively stable post-fusion, but long-term storage may induce phase separation.	Stable coating via native membrane–membrane interactions; high dissociation resistance.	A covalent bond forms a stable linkage with resistance to dissociation under physiological conditions.	The avidin–biotin system offers high stability; electrostatic adsorption is sensitive to pH/ionic strength.
Scalability	Well-established process, suitable for large-scale production.	Batch variability of membrane composition; complex scale-up.	Relatively mature but multi-step processes.	Standardized protocols feasible, but multi-step purification is required, leading to higher cost.	Simple process, readily scalable, suitable for industrial production.
Potential immunogenicity	No introduction of exogenous chemical groups; preserves native characteristics.	Lower intrinsic immunogenicity than mammalian-derived vesicles.	Autologous or homologous cell membrane camouflage reduces immunogenicity and enables immune evasion.	Introduction of non-natural linkers may generate neo-epitopes.	Exogenous proteins such as biotin/avidin may be immunogenic.
Regulatory readiness	Well-established physical loading method; the TIBS system enables standardized production.	Preclinical/early-stage; regulatory pathway undefined.	Clear potential for clinical translation.	Technologically mature, but covalently modified PDEVs have not yet entered the regulatory pathway.	Technology is mature in the field of bioconjugation, but regulatory experience for PDEV applications remains limited.
Structural integrity	Electroporation or sonication may induce irreversible membrane perforation.	Fusion may alternative membrane structure.	Mild extrusion; preserves core structure.	Chemical reaction conditions may damage surface proteins and lipids.	Chemical reaction conditions may damage surface proteins and lipids.
Reference	[92]	[93,94,95]	[96]	[93,95]	[94]

## Data Availability

Data sharing is not applicable to this article as no data were created or analyzed in this research.
